# Association of subchondral bone texture on magnetic resonance imaging with radiographic knee osteoarthritis progression: data from the Osteoarthritis Initiative Bone Ancillary Study

**DOI:** 10.1007/s00330-018-5444-9

**Published:** 2018-05-02

**Authors:** James W. MacKay, Geeta Kapoor, Jeffrey B. Driban, Grace H. Lo, Timothy E. McAlindon, Andoni P. Toms, Andrew W. McCaskie, Fiona J. Gilbert

**Affiliations:** 1 Department of Radiology, University of Cambridge, Cambridge Biomedical Campus, Box 218 Hills Road, Cambridge, CB2 0QQ UK; 2 Department of Radiology, Norfolk & Norwich University Hospitals NHS Foundation Trust, Colney Lane, Norwich, NR4 7UY UK; 3 Division of Rheumatology, Tufts Medical Center, 800 Washington Street, Boston, MA 02111 USA; 4 Department of Medicine, Baylor College of Medicine, 1 Baylor Plaza, BCM-285, Houston, TX 77030 USA; 5 Norwich Medical School, University of East Anglia, Norwich Research Park, Norwich, NR4 7TJ UK; 6 Division of Trauma and Orthopaedic Surgery, Department of Surgery, University of Cambridge, Cambridge Biomedical Campus, Box 180 Hills Road, Cambridge, CB2 0QQ UK

**Keywords:** Osteoarthritis, knee, Magnetic resonance imaging, Case-control studies, Logistic models

## Abstract

**Objectives:**

To assess whether initial or 12–18-month change in magnetic resonance imaging (MRI) subchondral bone texture is predictive of radiographic knee osteoarthritis (OA) progression over 36 months.

**Methods:**

This was a nested case-control study including 122 knees/122 participants in the Osteoarthritis Initiative (OAI) Bone Ancillary Study, who underwent MRI optimised for subchondral bone assessment at either the 30- or 36-month and 48-month OAI visits. Case knees (n = 61) had radiographic OA progression between the 36- and 72-month OAI visits, defined as ≥ 0.7 mm minimum medial tibiofemoral radiographic joint space (minJSW) loss. Control knees (n = 61) without radiographic OA progression were matched (1:1) to cases for age, sex, body mass index and initial medial minJSW. Texture analysis was performed on the medial femoral and tibial subchondral bone. We assessed the association of texture features with radiographic progression by creating a composite texture score using penalised logistic regression and calculating odds ratios. We evaluated the predictive performance of texture features for predicting radiographic progression using *c*-statistics.

**Results:**

Initial (odds ratio [95% confidence interval] = 2.13 [1.41–3.40]) and 12– 18-month change (3.76 [2.04–7.82]) texture scores were significantly associated with radiographic OA progression. Combinations of texture features were significant predictors of radiographic progression using initial (*c*-statistic [95% confidence interval] = 0.65 [0.64–0.65], *p* = 0.003) and 12–18-month change (0.68 [0.68-0.68], *p* < 0.001) data.

**Conclusions:**

Initial and 12–18-month changes in MRI subchondral bone texture score were significantly associated with radiographic progression at 36 months, with better predictive performance for 12–18-month change in texture. These results suggest that texture analysis may be a useful biomarker of subchondral bone in OA.

**Key Points:**

*• Subchondral bone MRI texture analysis is a promising knee osteoarthritis imaging biomarker.*

*• In this study, subchondral bone texture was associated with knee osteoarthritis progression.*

*• This demonstrates predictive and concurrent validity of MRI subchondral bone texture analysis.*

*• This method may be useful in clinical trials with interventions targeting bone.*

**Electronic supplementary material:**

The online version of this article (10.1007/s00330-018-5444-9) contains supplementary material, which is available to authorized users.

## Introduction

It is increasingly recognised that subchondral bone plays a critical role in osteoarthritis (OA) onset and progression. Subchondral bone is a dynamic tissue, absorbing the majority of forces transmitted through the joint and capable of remodelling in response to stress [1]. Therefore, there has been increasing interest in subchondral bone as a target for potential disease-modifying OA drugs (DMOADs) [2].

Sensitive markers of subchondral bone alterations that occur in OA are required for such treatments to be evaluated. Several imaging biomarkers of subchondral bone have been described using plain radiographs, dual x-ray absorptiometry (DXA), computed tomography (CT) and magnetic resonance imaging (MRI), including direct estimation of trabecular microarchitecture and fractal signature analysis (FSA) [3, 4]. While several established biomarkers have shown cross-sectional associations with OA severity, there remains room for improvement with regard to the ability to predict OA progression [5–8].

MRI texture analysis (MRI TA) has recently been described as a method of quantifying subchondral bone changes in OA which may offer superiority over existing biomarkers [9]. MRI TA involves the calculation of several statistical descriptors of image texture, aiming to characterise the heterogeneity and spatial-organisation of the subchondral bone. The technique has been shown to be reproducible and able to distinguish subjects at different stages of OA from healthy subjects [9]. Moreover, texture features have shown an improved ability to discriminate between knees with and without OA when compared to microarchitectural analysis and are significantly associated with histomorphometry [10, 11].

However, MRI TA has previously only been used to compare subjects with OA and healthy controls. To be useful as a prognostic or treatment evaluation imaging biomarker, it should also be able to identify which individuals with OA are likely to progress (predictive validity) and demonstrate sensitivity to change for OA progression (concurrent validity).

Therefore, the purpose of this study was to evaluate whether (1) initial (i.e. OAI visit at 30 or 36 months) or (2) 12–18-month change (i.e. between OAI visit at 30/36 months and 48 months) in MRI subchondral bone texture were predictive of radiographic OA progression over 36 months (i.e. between OAI visit at 36 months and 72 months).

## Methods

The Osteoarthritis Initiative (OAI) has been approved by the Institutional Review Boards of the University of California, San Francisco and the four OAI clinical centers (University of Pittsburgh, Ohio State University, University of Maryland, Baltimore, and Memorial Hospital of Rhode Island). All participants have given informed consent to participate in the study. This was a retrospective nested case-control study. The OAI datasets are freely available for download at https://oai.epi-ucsf.org.

### Subjects

**Fig. 1:**
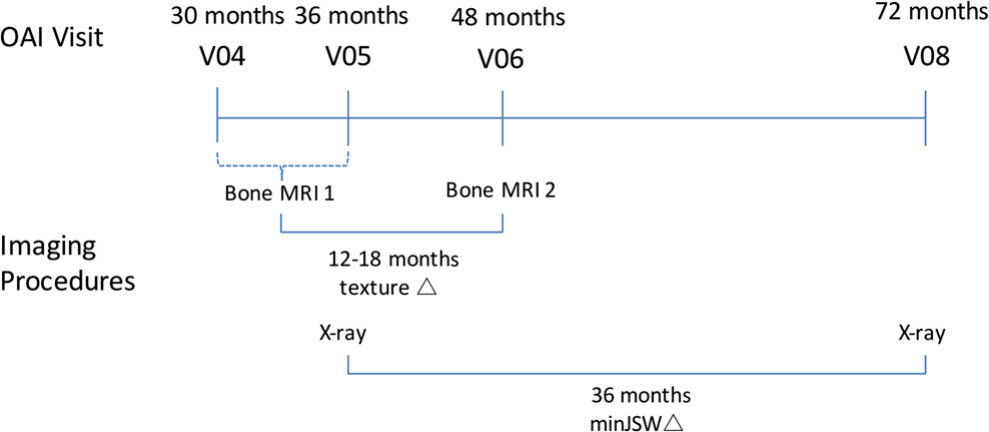
Study timeline for image assessments as part of the Osteoarthritis Initiative

Included participants were participants in the OAI Bone Ancillary Study (BAS). The BAS featured 629 participants who underwent MRI examination optimised for assessment of subchondral bone in addition to the standard OAI MRI. All BAS participants were members of the progression sub-cohort, participants with both frequent knee symptoms and radiographic OA in at least one knee at OAI inception. Initial trabecular bone MRIs were performed at either the 30- or 36-month OAI visit, with the majority of participants undergoing a repeat MRI at the 48-month OAI visit (“12–18-month follow-up”). The study design is summarised in Fig. 1.

We defined study cases (“progressors”) as individuals with radiographic progression over a 36-month period between the 36-month OAI visit and the 72-month OAI visit, according to the definition of the Foundation for the National Institutes of Health (FNIH) OA biomarkers consortium: a decrease in minimum medial tibiofemoral joint space width (minJSW) of ≥ 0.7 mm, which has a less than 10% chance of being due to measurement error [12]. The details of the radiographic acquisition and assessments have been discussed in detail previously. Briefly, knee radiographs were performed using a non-fluoroscopic fixed flexion technique [13]. Assessments included central readings for Kellgren-Lawrence (KL) grading, Osteoarthritis Research Society International (OARSI) grading of joint space narrowing (JSN), measurement of femorotibial alignment and automated measurement of joint space width [14–16].

Progressors were matched to participants who did not have radiographic progression between the 36-month OAI visit and 72-month OAI visit (controls) for age, sex, body mass index (BMI) and initial medial minJSW in a 1:1 ratio, using an optimal nearest-neighbour propensity score algorithm.

We excluded individuals who did not have measurements of minJSW available at the 36-month or 72-month OAI visits and individuals with KL grade-4 knees at the 36-month visit (due to ceiling effects on minJSW progression in this group). As this study focused on the medial tibiofemoral compartment, we excluded any individuals who had lateral compartment predominant disease at the 36-month OAI visit, as defined by greater OARSI JSN grade in the lateral than medial compartment.

### MRI acquisition

Study participants were evaluated with a coronal-oblique three-dimensional fast imaging with steady-state precession (FISP) MRI sequence (field of view 12 x 12 cm, matrix 512 x 512 [interpolated to 1024 x 1024], slice thickness 1 mm, repetition time 20 ms, echo time 4.92 ms, flip angle 50^o^, number of signal averages 1, acquisition time 10.5 minutes) optimised for visualisation of subchondral trabecular bone [17]. This was performed on one of four identical Siemens Trio 3T MR platforms used for the OAI using a quadrature transmit-receive knee coil (USA Instruments).

### MRI analysis

The five most central coronal-oblique images through the central medial tibiofemoral joint were identified with reference to axial and sagittal reformats and used for subsequent analysis.

**Fig. 2:**
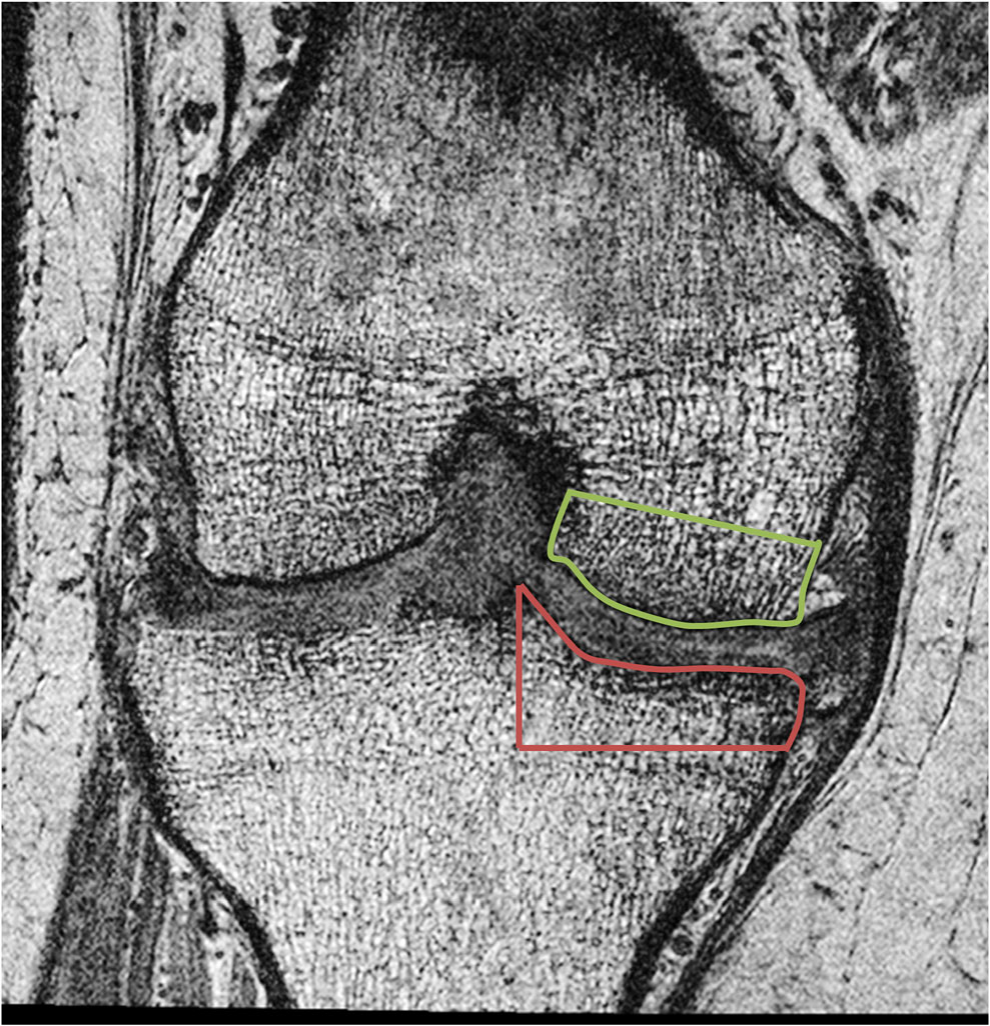
Example coronal-oblique 3D FISP MR image demonstrating ROI placement in the medial tibial (red) and medial femoral (green) subchondral bone

The MRI images were imported into a dedicated texture analysis program (MazDA v3.3, freely available at http://www.eletel.p.lodz.pl/programy/mazda/) [18]. Regions of interest (ROIs) were created manually in the medial tibial and medial femoral subchondral bone on each coronal image by an analyst blinded to case or control status of participants (JM, a musculoskeletal radiologist with 5 years’ experience). ROIs were defined superiorly and inferiorly by the bone–cartilage interface, medially and laterally by the margins of the tibial plateau and femoral condyle and extended for a depth of approximately 1 cm into the subchondral bone. Illustrative ROI examples are provided in Fig. 2.

Nineteen texture features were calculated for each ROI aiming to quantify the heterogeneity and spatial organisation of the subchondral bone, according to the method described previously [9]. These texture features belonged to one of four classes: grey-level histogram, absolute gradient, run-length matrix (RLM) and grey-level co-occurrence matrix (GLCM). Briefly, grey-level histogram features are simple descriptors of the distribution of grey levels (i.e. pixel intensity values) in the ROI. Gradient, RLM and GLCM features are higher-order descriptors of the spatial organisation of pixels in the ROI. We used image compression settings of four bits/pixel for calculation of gradient features, and six bits/pixel for calculation of GLCM and RLM parameters. RLM parameters were calculated four times for each pixel (in the horizontal, vertical, 45^o^ and 135^o^ directions) and GLCM parameters were calculated 20 times for each pixel at a variety of pixel offsets ranging from 1 to 5 pixels. The mean value of each RLM and GLCM parameter for each pixel in all possible directions and pixel offsets was calculated for each coronal image. The values of each texture parameter on each of the five coronal images analysed were then averaged to give summary values in each participant for medial tibial and medial femoral ROIs.

### Texture analysis reproducibility

Twenty-three randomly selected participants were analysed in duplicate by two independent analysts (JM & GK: both musculoskeletal radiologists with 5 years’ experience) to assess reproducibility. Analysts created ROIs independently and were blinded to case or control status. The sample size was based on previous data, suggesting a mean intraclass correlation coefficient (ICC) value of 0.9 across texture features [10, 19]. Texture features with suboptimal reproducibility metrics (ICCs of < 0.8 or root-mean-square average coefficient of variation [RMSCV] of > 10% for either ROI) were excluded from subsequent analyses.

### Statistical analysis

Descriptive statistics for each texture feature were generated. We compared the distribution of texture values in progressors and controls visually using boxplots. We created composite texture scores using linear combinations of texture features with least absolute shrinkage and selection operator (LASSO) penalised logistic regression. This helps to avoid problems associated with overfitting when many predictor variables are available by imposing a penalty for including additional variables in a model. The LASSO model was chosen based on minimisation of 10-fold cross-validation classification error. Because the folds for cross-validation are chosen at random, the procedure was repeated with 100 iterations. We used the logistic regression equation from the model iteration giving the mean classification error to calculate a composite texture score. This was performed separately for tibial, femoral and combined (tibial and femoral) texture datasets using initial MRI texture features and 12–18-month change in MRI texture features.

Odds ratios were calculated for the increase or decrease in odds of radiographic progression per one standard deviation (SD) increase in texture score. The mean classification accuracy (*c*-statistic, equivalent to area under the receiver operating curve [AUC]) was recorded.

Inter-observer reproducibility was assessed using ICC values (single measures, absolute agreement) and the RMSCV for each texture feature.

All statistical analyses were performed using RStudio version 1.0.136 for Mac, using the *MatchIt* package for matching cases to controls, and the *glmnet* package for performing LASSO regression [20, 21]. Statistical significance of the logistic regression analyses was assessed using the chi-squared test, with an adjusted *p* value threshold of < 0.008 to maintain an overall type 1 error rate of 0.05.

## Results

### Participants

Of 629 participants in the BAS, 359 were eligible for this study following exclusions. Sixty-four participants met the criterion for radiographic progression and were selected as cases, with 64 controls matched for age, sex, BMI and initial minJSW.

For initial timepoint analyses, 12 participants (3 cases) were excluded due to excessive motion artefacts, defined as preventing the identification of the bone–cartilage interface (n = 5), unavailable initial MRI (n = 3) or large subchondral cysts preventing ROI placement in trabecular bone (n = 4). The matched controls for excluded cases were also removed from the analysis. For each excluded control, a new matched control was selected as the next best match for the corresponding case according to the matching algorithm.

For 12–18-month follow-up analyses, a further 17 participants were excluded due to excessive motion artefacts on follow-up images (n = 8), unavailable follow-up MRI (n = 8), and surgical intervention during the interval between initial and follow-up MRI (n = 1).

**Fig. 3:**
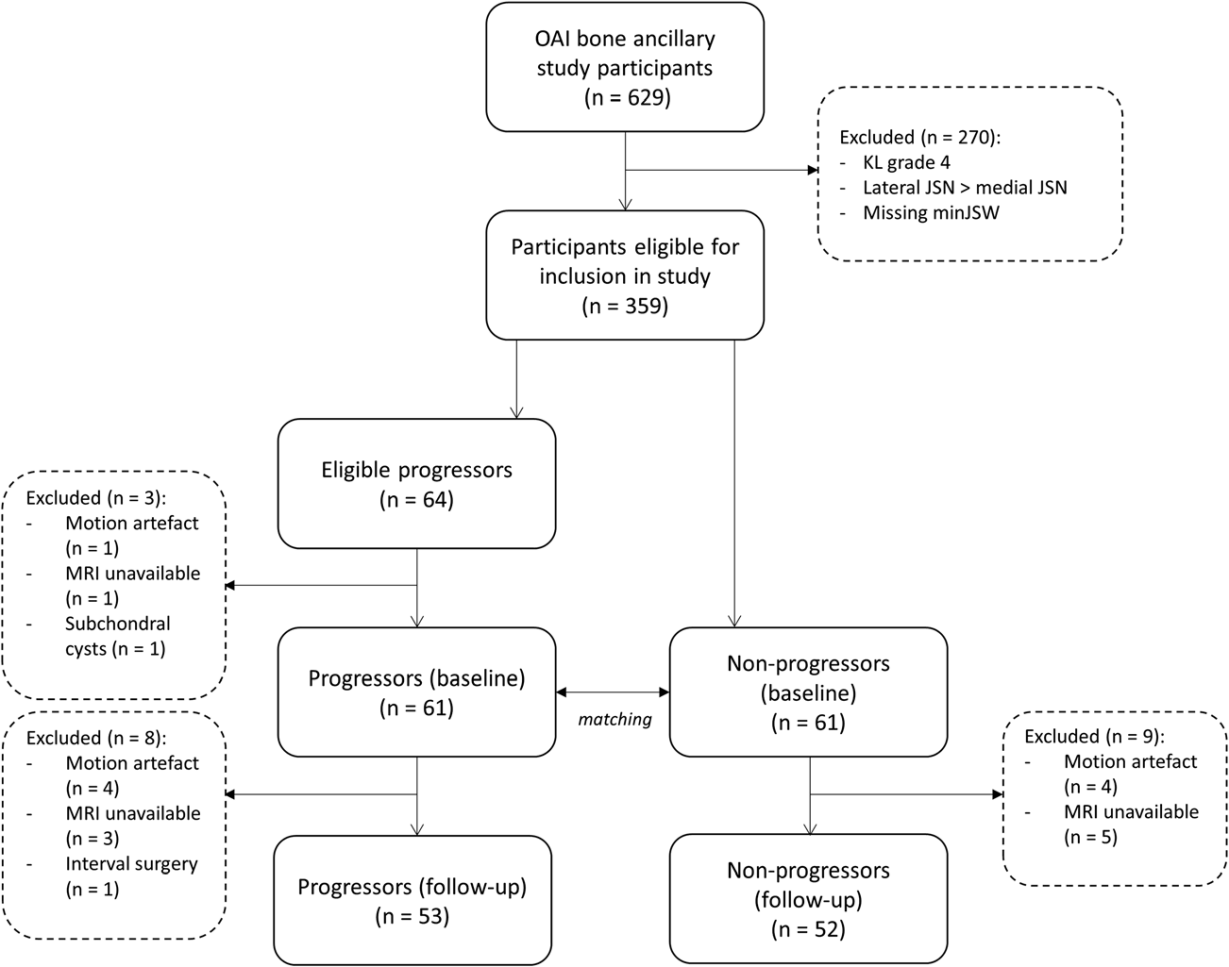
Flow diagram for selection of study participants

Initial and follow-up characteristics of included cases and controls are presented in Table 1. A flow diagram for selection of study participants is presented in Fig. 3.

**Table 1:** Participant characteristics at initial timepoint and 12–18-month follow-up

Variable	Initial		12–18-Month follow-up	
	Cases	Controls	Cases	Controls
	n = 61	n = 61	n = 53	n = 52
Age, years^a^	64 (49–81)	65 (48–82)	65 (50–82)	66 (49–83)
Sex, no. females	25	26	21	22
BMI, kg/m^2 b^	31.4 (4.7)	31.1 (4.7)	31.1 (4.5)	30.8 (4.6)
Time between baseline and follow-up MRI (12/18 months)	–	–	35/18	26/26
Initial minJSW, mm^b^	3.81 (1.20)	3.78 (1.19)	3.86 (1.19)	3.75 (1.20)
Kellgren Lawrence grade (0/1/2/3)	7/11/21/22	8/9/24/20	7/11/17/18	8/9/20/15
OARSI medial JSN grade (0/1/2)	17/22/22	23/18/20	15/20/18	21/16/15
OARSI lateral JSN grade (0/1/2)	60/1/0	58/3/0	52/1/0	50/2/0
Femorotibial alignment, degrees^c^	–6.0 (1.9)	–5.7 (2.1)	–6.0 (1.8)	–5.7(2.1)
minJSW change, mm^b^	–	–	–1.29 (0.63)	0 (0.44)

^a^Mean (range)

^b^Mean (standard deviation)

^c^Mean (standard deviation), negative values indicate varus alignment

### Texture analysis reproducibility

Data for inter-observer reproducibility are presented in the supplementary material. The majority of texture features demonstrated excellent inter-observer reproducibility. Seven texture features were excluded from subsequent analyses at this stage (per criteria in *Methods*), leaving a total of 12 texture features for analysis.

### Association of subchondral bone texture and radiographic progression

**Fig. 4:**
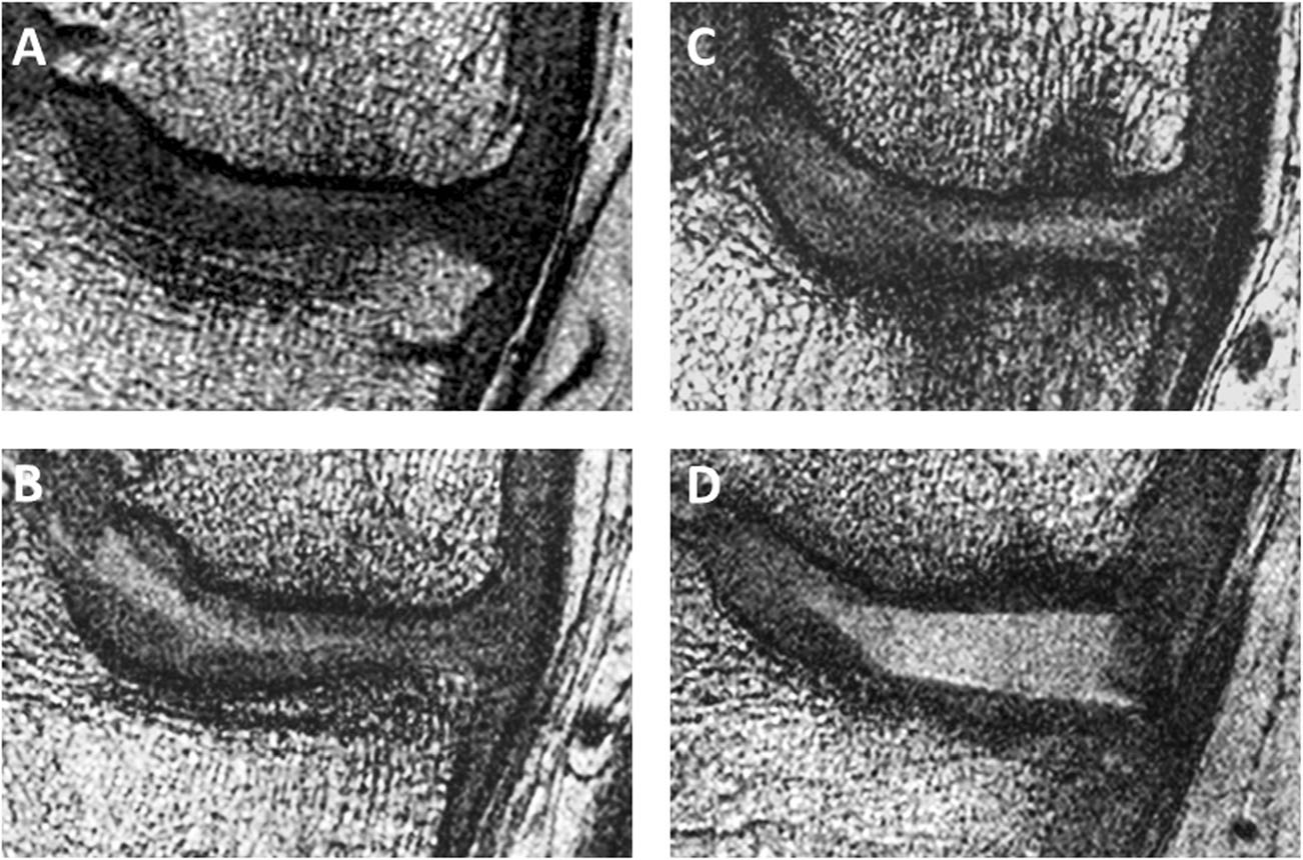
Example coronal 3D FISP MR images through the medial tibiofemoral compartment of radiographic progressors and non-progressors at the initial timepoint. *A*, non-progressor, texture score (TS) –1.22, *B*, non-progressor, TS –1.44, *C*, progressor, TS +1.01, and *D*, progressor, TS +0.93. Higher texture scores correspond to less spatially organised subchondral bone

Subchondral bone composite texture score was significantly associated with 36-month radiographic progression using the initial timepoint combined data (odds ratio [95% confidence interval] = 1.84 [1.25 – 2.80], *p* = 0.002) and the 12–18-month change tibial (2.31 [1.42 – 4.12], *p* < 0.001), femoral (1.80 [1.17 – 2.92], *p* = 0.006) and combined (3.76 [2.04 – 7.82], *p* < 0.001) data. Associations between subchondral bone texture score and radiographic progression using initial timepoint tibial (1.43 [0.99 – 2.09], *p* = 0.06) and femoral (1.63 [1.12 – 2.44], *p* = 0.009) data were not statistically significant. Results are summarised in Table 2. Data for each individual texture feature are provided in the supplementary material. Example images are shown in Fig. 4.

**Table 2:** Association between texture features and case vs. control status and classification performance

Region	Initial			12–18-Month change		
	Odds ratio (95% CI)^a^	Most important features^b^	*c*-statistic (95% CI)	Odds ratio (95% CI)^a^	Most important features^b^	*c*-statistic (95% CI)
Tibia	1.43 (0.99, 2.09)	Gr. variance, variance	0.58 (0.58, 0.58)	2.31 (1.42, 4.12)***	Gr. mean, Gr. variance, contrast	0.65 (0.63, 0.69)**
Femur	1.62 (1.12, 2.44)**	Mean, variance, ASM	0.60 (0.59, 0.60)*	1.80 (1.17, 2.92)**	ASM, contrast, variance	0.63 (0.61, 0.65)**
Combined	1.84 (1.25, 2.80)**	F variance, F mean, T variance	0.64 (0.64, 0.65)**	3.76 (2.04, 7.82)***	T Gr. variance, F ASM, T entropy	0.68 (0.68, 0.68)***

T – tibial feature, F – femoral feature, Gr – gradient, ASM – angular second moment

* *p* < 0.05, ** *p* < 0.01, ***p < 0.001

^a^Odds ratio of being a progressor for each o standard deviation increase in texture score

^b^Three texture features with largest standardised coefficients (β) in the logistic regression model (note initial tibial model only included two texture features)

### Texture analysis classification

Combinations of both initial texture features and 12–18-month change in texture features were able to predict radiographic progression with statistical significance reached for femoral and combined initial data, and tibial, femoral and combined 12–18-month change data. The best classification accuracy was demonstrated for combined 12–18-month follow-up data with a *c*-statistic of 0.68 (95% confidence interval 0.68 – 0.68, *p* < 0.001).

Classification performance is summarised in Table 2.

## Discussion

This study demonstrates that combinations of initial and 12–18-month change in subchondral bone MR texture features are significantly associated with radiographic OA progression over 36 months, with better predictive ability for 12–18-month change data.

These results suggest that subchondral bone texture may be a useful quantitative imaging biomarker for use in clinical trials, particularly those with interventions targeting subchondral bone. Although direct interpretation of the texture scores used in this study is difficult, results are consistent with progressors having less spatially organised, more homogeneous subchondral bone at the initial timepoint, and 12–18-month changes in the same direction. This is supported by visual assessment of example images. Combinations of texture features were able to predict case and control status using both initial and 12–18-month change in texture features. Classification accuracy improved when tibial and femoral data were combined. However, the best-performing model had only modest predictive ability (AUC 0.68), despite the strong associations between texture score and radiographic progression.

Possible explanations for the limited performance of MR subchondral bone texture in this study include a bias towards advanced OA in our cohort, and the MRI sequence used. First, as members of the progression sub-cohort of the OAI, most participants had established OA. Our study sample was further biased towards more advanced disease by the fact that only individuals with established OA at the 48-month OAI visit had quantitative JSW measurements performed at the 72-month OAI visit. MR subchondral bone texture has previously demonstrated the ability to detect relatively early OA-related changes in the subchondral bone; therefore, it may be that it is of less use in more established disease. Second, the MRI sequence used as part of the OAI BAS was optimised for analysing trabecular microarchitecture (i.e. direct estimation of histomorphometry parameters). While texture analysis has previously been described using a similar MRI sequence, it has also been demonstrated that alternative MRI sequences may provide improved texture discrimination between groups [22].

Nevertheless, the strength of association between texture scores and radiographic progression and the AUC values presented here are competitive when compared to several alternative OA imaging biomarkers, especially considering the relatively short follow-up time and matching of the control cohort in this study for important predictive covariates of age, sex and BMI [5, 23–26]. For example, in the FNIH OA biomarkers consortium studies, central medial tibiofemoral compartment cartilage loss over 24 months was associated with radiographic progression at 24–28 months with a similar odds ratio (3.8 [95% CI 2.7 to 5.5]) to that for 12–18-month change in combined texture score (3.8 [2.0 – 7.8]) in this study [25]. In the same FNIH cohort, associations between femoral bone shape change (odds ratio 2.7 [2.0 to 3.6]) and femoral bone area change (2.9 [2.1 to 3.9]) over 24 months and radiographic progression at 24-48 months were also of a similar magnitude to those described here [26]. Associations between fractal signature analysis (FSA) parameters obtained from plain radiographs and radiographic progression in the FNIH OA biomarkers cohort were weaker than those described in this study, as was the predictive ability of FSA [5].

Our results demonstrate that MRI TA of subchondral bone can be considered a useful addition to the suite of imaging biomarkers available for further OA imaging research studies. One advantage over alternative techniques is the multidimensional data output of texture analysis, which is well-placed to interact with machine-learning-based approaches to image interpretation. Indeed, the LASSO method employed here is an example of such an approach.

One disadvantage of MRI TA is that it is not always clear what the biological or structural correlates of individual texture features are, despite previous demonstration of association with histomorphometry [11]. However, as has been shown by the use of texture analysis in imaging applications other than assessment of subchondral bone, this lack of correspondence to an underlying structural ‘ground-truth’ does not preclude the use of this method to improve our understanding of the underlying disease process [27–29]. In the present study, MRI TA has usefully quantified the degree of ‘abnormality’ in the appearance of the subchondral bone despite limited structural correlation of the texture parameters used.

Future work could evaluate MRI TA of subchondral bone in alternative populations, and a head-to-head comparison of different methods for analysing subchondral bone would help to determine the optimal imaging biomarker for use in clinical trials. A barrier to performing this comparison in the present study was the fact that the platforms used for several alternative methods are not freely available, in contrast to the method used here. Automation of the time-consuming ROI-drawing procedure would also encourage wider use of this method.

There are several limitations to the present study. Structural correlates for texture parameters have been assessed in previous studies, but using a different MRI sequence. Nevertheless, a comparison of texture features between sequences has shown similar changes in both, so it is reasonable to assume similar structural correlates for the texture features derived from the MRI sequence used in this study [22]. We used a retrospective case-control design which is subject to selection bias. However, our matching process ensured that cases and controls were well-matched for important baseline characteristics. In common with other longitudinal studies using the OAI dataset, it is not possible to completely separate concurrent from predictive validity for MR subchondral bone texture as the period of follow-up for change in MR texture features overlapped with the follow-up period for radiographic progression. We had a small sample size for the number of texture features analyzed which risks introducing bias into any classification procedure. We have aimed to minimise this by excluding texture features with poor reproducibility, using cross-validation, and using penalised regression to limit the number of texture features incorporated into our classification models. Finally, we defined OA progression based on change in radiographic joint space width. This measure is established and robust but captures only one aspect of OA progression. Symptomatic progression is also important, but not considered in this study due to the low numbers of symptomatic progressors in our cohort.

In conclusion, initial and 12–18-month change in combinations of MR subchondral bone texture features were associated with 36-month radiographic OA progression, with better predictive performance of 12–18-month change data.

## Electronic supplementary material

ESM 1 (DOCX 34 kb)

## Abbreviations

BAS: Bone Ancillary Study
OAI: Osteoarthritis Initiative
minJSW: Minimum (medial tibiofemoral) joint space width
MRI TA: Magnetic resonance imaging texture analysis
