# Supplementary material for: Association of subchondral bone texture on magnetic resonance imaging with radiographic knee osteoarthritis progression: data from the Osteoarthritis Initiative Bone Ancillary Study
Source: Eur Radiol. 2018 May 2;28(11):4687–95. doi: 10.1007/s00330-018-5444-9 (PMC6182744; doi:10.1007/s00330-018-5444-9)
Supplement: Supplementary file 1 — (DOCX 34 kb) [file 330_2018_5444_MOESM1_ESM.docx]

### Supplementary Table 1. Inter-observer agreement data for individual subchondral bone texture features.

| **Parameter** | **ICC (95% CI)^a^** | **RMS-CV (%)** |
| --- | --- | --- |
| *Tibia* |  |  |
| **Mean** | **0.95 (0.90-0.98)** | **5.5** |
| Skewness | 0.78 (0.56-0.90) | 24.4 |
| Kurtosis | 0.69 (0.40-0.86) | 43.4 |
| **Variance** | **0.94 (0.87-0.97)** | **5.5** |
| **Gradient Mean** | **0.97 (0.94-0.99)** | **1.8** |
| Gradient Skewness | 0.90 (0.77-0.95) | 10.9 |
| Gradient Kurtosis | 0.63 (0.30-0.82) | 42.4 |
| **Gradient Variance** | **0.98 (0.96-0.99)** | **1.4** |
| **Gradient NonZeros** | **0.93 (0.83-0.97)** | **0.9** |
| **Run fraction** | **0.95 (0.88-0.98)** | **0.4** |
| **Short run-length emphasis** | **0.94 (0.87-0.98)** | **0.3** |
| **Long run-length emphasis** | **0.93 (0.85-0.97)** | **1.1** |
| Grey-level non-uniformity | 0.62 (0.29-0.82) | 10.0 |
| Run-length non-uniformity | 0.59 (0.25-0.80) | 13.6 |
| **Angular Second Moment** | **0.88 (0.75-0.95)** | **8.2** |
| **Contrast** | **0.98 (0.95-0.99)** | **3.9** |
| Correlation | 0.75 (0.47-0.89) | 4.0 |
| **Entropy** | **0.94 (0.87-0.98)** | **1.1** |
| **Inverse difference moment** | **0.95 (0.88-0.98)** | **2.2** |
| *Femur* |  |  |
| **Mean** | **0.95 (0.89-0.98)** | **3.8** |
| Skewness | 0.88 (0.73-0.95) | 18.8 |
| Kurtosis | 0.92 (0.03-0.97) | 43.1 |
| **Variance** | **0.97 (0.92-0.99)** | **5.3** |
| **Gradient Mean** | **0.98 (0.94-0.99)** | **1.6** |
| Gradient Skewness | 0.96 (0.92-0.98) | 14.3 |
| Gradient Kurtosis | 0.89 (0.77-0.95) | 17.5 |
| **Gradient Variance** | **0.98 (0.96-0.99)** | **2.1** |
| **Gradient NonZeros** | **0.95 (0.88-0.98)** | **0.7** |
| **Run fraction** | **0.96 (0.91-0.98)** | **0.3** |
| **Short run-length emphasis** | **0.96 (0.91-0.98)** | **0.2** |
| **Long run-length emphasis** | **0.95 (0.89-0.98)** | **1.0** |
| Grey-level non-uniformity | 0.80 (0.58-0.91) | 9.8 |
| Run-length non-uniformity | 0.66 (0.35-0.84) | 11.7 |
| **Angular Second Moment** | **0.95 (0.88-0.98)** | **4.2** |
| **Contrast** | **0.98 (0.95-0.99)** | **3.6** |
| Correlation | 0.81 (0.60-0.91) | 8.0 |
| **Entropy** | **0.96 (0.92-0.98)** | **0.8** |
| **Inverse difference moment** | **0.96 (0.91-0.98)** | **1.8** |

^a^single measures, absolute agreement

Texture features in **bold** were included in subsequent analyses

Abbreviations: ICC – intraclass correlation coefficient, RMS-CV – root mean square average of the coefficients of variation, Gradient NonZeros – proportion of pixels with non-zero gradient

**Supplementary Table 2.** Initial and 12-18 month change in subchondral bone texture in case and control knees

| Texture feature | Baseline | | | 12-18 month change | | |
| --- | --- | --- | --- | --- | --- | --- |
|  | Cases  (mean (SD)) | Controls (mean (SD)) | OR (95% CI)^a^ | Cases  (mean (SD)) | Controls (mean (SD)) | OR (95% CI)^a^ |
| *Tibia* |  |  |  |  |  |  |
| Mean | 178 (40) | 191 (35) | 0.77 (0.53 – 1.11) | 11.8 (42.4) | 3.7 (40.5) | 1.22 (0.83 – 1.87) |
| Variance | 8060 (2890) | 9000 (3112) | 0.73 (0.50 – 1.04) | 902 (3198) | 501 (2688) | 1.15 (0.78 – 1.71) |
| GrMean | 1.63 (0.36) | 1.68 (0.36) | 0.88 (0.61 – 1.26) | 0.04 (0.49) | 0.12 (0.46) | 0.84 (0.57 – 1.24) |
| GrVariance | 0.95 (0.34) | 0.99 (0.35) | 0.89 (0.61 – 1.27) | 0.05 (0.48) | 0.10 (0.40) | 0.89 (0.60 – 1.31) |
| GrNonZeros | 0.88 (0.05) | 0.88 (0.06) | 0.91 (0.63 – 1.30) | 0.004 (0.06) | 0.02 (0.08) | 0.80 (0.53 – 1.18) |
| Run Fraction | 0.87 (0.03) | 0.87 (0.03) | 0.90 (0.63–1.29) | 0.003 (0.04) | 0.01 (0.04) | 0.81 (0.54 – 1.19) |
| SRLE | 0.90 (0.02) | 0.90 (0.02) | 0.90 (0.62 – 1.29) | 0.003 (0.03) | 0.01 (0.03) | 0.81 (0.54 0 1.19) |
| LRLE | 1.52 (0.16) | 1.51 (0.18) | 1.08 (0.76 – 1.56) | -0.01 (0.20) | -0.06 (0.23) | 1.24 (0.84 – 1.87) |
| ASM^b^ | 3.93 (1.93) | 3.72 (2.10) | 1.11 (0.78 – 1.61) | -0.25 (2.17) | -0.92 (2.68) | 1.33 (0.90 – 2.03) |
| Contrast | 38.3 (18.0) | 40.5 (17.9) | 0.88 (0.61 – 1.26) | 2.3 (25.3) | 5.8 (21.6) | 0.86 (0.58 – 1.26) |
| Entropy | 2.58 (0.20) | 2.61 (0.20) | 0.88 (0.61 – 1.25) | 0.03 (0.25) | 0.09 (0.25) | 0.80 (0.54 – 1.18) |
| Inv Df Mom | 0.22 (0.04) | 0.21 (0.04) | 1.13 (0.79 – 1.63) | -0.005 (0.05) | -0.02 (0.06) | 1.25 (0.85 – 1.86) |
| *Femur* |  |  |  |  |  |  |
| Mean | 176 (34) | 187 (33) | 0.70 (0.47 – 1.00) | 13.8 (36.5) | 8.3 (28.4) | 1.18 (0.80 – 1.78) |
| Variance | 6940 (2677) | 7433 (2122) | 0.87 (0.60 – 1.24) | 1086 (2440) | 551 (2221) | 1.27 (0.86 – 1.93) |
| GrMean | 1.67 (0.36) | 1.72 (0.38) | 0.89 (0.62 – 1.27) | 0.07 (0.45) | 0.17 (0.48) | 0.80 (0.53 – 1.18) |
| GrVariance | 0.97 (0.35) | 1.00 (0.36) | 0.92 (0.64 – 1.31) | 0.06 (0.48) | 0.14 (0.45) | 0.82 (0.55 – 1.22) |
| GrNonZeros | 0.89 (0.05) | 0.89 (0.06) | 0.94 (0.65 – 1.34) | 0.009 (0.05) | 0.03 (0.07) | 0.78 (0.51 – 1.15) |
| Run Fraction | 0.87 (0.03) | 0.88 (0.03) | 0.92 (0.64 – 1.31) | 0.006 (0.03) | 0.003 (0.04) | 0.78 (0.52 – 1.15) |
| SRLE | 0.90 (0.02) | 0.91 (0.03) | 0.92 (0.64 – 1.31) | 0.004 (0.02) | 0.003 (0.03) | 0.77 (0.51 – 1.14) |
| LRLE | 1.51 (0.15) | 1.50 (0.18) | 1.06 (0.74 – 1.53) | -0.03 (0.16) | -0.08 (0.23) | 1.29 (0.87 – 1.96) |
| ASM^b^ | 4.27 (2.35) | 4.27 (2.70) | 1.00 (0.70 – 1.44) | -0.45 (2.24) | -1.31 (3.34) | 1.37 (0.92 – 2.12) |
| Contrast | 38.6 (17.5) | 40.5 (18.6) | 0.90 (0.62 – 1.29) | 3.0 (23.8) | 8.1 (22.8) | 0.80 (0.53 – 1.18) |
| Entropy | 2.55 (0.20) | 2.56 (0.22) | 0.93 (0.65 – 1.34) | 0.05 (0.24) | 0.11 (0.28) | 0.78 (0.52 – 1.15) |
| Inv Df Mom | 0.21 (0.04) | 0.21 (0.05) | 1.10 (0.77 – 1.59) | -0.009 (0.05) | -0.005 (0.05) | 1.30 (0.88 – 1.97) |

^a^ odds ratio (OR) and 95% confidence interval (95% CI) of being a case for a 1 standard deviation increase in texture feature

^b^ values as given x10-3

**Abbreviations:** Gr – Gradient, GrNonZeros – proportion of pixels with non-zero gradient, SRLE – short run-length emphasis, LRLE – long run length emphasis, ASM – angular second moment, Inv Df Mom – inverse difference moment.
